# Assessment of Health-Related Quality of Life Using EuroQoL-5 Dimension in Populations With Prediabetes, Diabetes, and Normal Glycemic Levels in Southwest China

**DOI:** 10.3389/fpubh.2021.690111

**Published:** 2021-10-12

**Authors:** Enwu Long, Shuang Feng, Li Zhou, Jie Chen, Lizheng Shi, Xuehua Jiang, Ming Hu, Nan Yang

**Affiliations:** ^1^West China School of Pharmacy, Sichuan University, Chengdu, China; ^2^Sichuan Academy of Medical Sciences and Sichuan Provincial People's Hospital, Chengdu, China; ^3^Department of Global Health Management and Policy, School of Public Health and Tropical Medicine, Tulane University, New Orleans, LA, United States

**Keywords:** diabetes, health-related quality of life (HRQoL), EuroQoL 5D (EQ-5D), EuroQol visual analog scale (EQ-VAS), propensity score (PS) matching (PSM)

## Abstract

**Objectives:** This study aimed to describe and compare health-related quality of life (HRQoL) among populations with normal glycemic levels, prediabetes, and diabetes in southwest China and to offer baseline data that can be easily compared to other regions in China or across countries.

**Methods:** A quality of life survey based on the EuroQoL-5 Dimension-5 level (EQ-5D-5L) scale was conducted through face-to-face or telephone interviews. A total of 403 respondents with diabetes, 404 with prediabetes, and 398 with normal blood glucose were enrolled in the survey. Propensity score matching (PSM) was used to decrease the bias of three groups, conditioned on age and gender, body mass index (BMI), and household income. For the three groups, we matched two groups first and then matched the result with the third group. Differences among groups were compared by chi-square test one-way ANOVA after adjusting by PSM.

**Results:** In general, the blood glucose of people with diabetes was generally well-controlled in southwest China, but they were often accompanied by the circulatory system and nutritional metabolic diseases. Ninety-nine individuals from each group were matched. The EuroQoL-5 Dimension index of the population with normal glycemic levels, prediabetes, and patients with diabetes was 0.901, 0.948, and 0.897. The EuroQol-visual analog scales (EQ-VAS) scores of each group above were 73.76, 77.45, and 68.34. HRQoL in males was higher than that of females in the three study groups. The results after PSM were consistent with that before matching.

**Conclusion:** There was a general trend that patients were associated with a decline of HRQoL from the prediabetic population, population with normal glycemic levels to diabetic population. Pain/discomfort and anxiety/depression might not be specific for the population with or without diabetes.

## Introduction

Diabetes is one of the most common and serious chronic diseases worldwide. An estimated 463 million adults were living with the condition in 2019, with numbers expected to reach 700 million by 2045 (1). China has become the county with the largest number (116.4 million) of adults with diabetes in the world, which is projected to exceed 147.2 million in 2045 (1). Prediabetes is defined as glycemic variables higher than normal, but lower than the threshold for diabetes, indicating a potential transition from normal glucose tolerance (NGT) to diabetes mellitus, and a relatively high risk of developing diabetes in the future (2). Without timely intervention, individuals with prediabetes frequently develop diabetes within 10 years (3). A nationally representative survey conducted in 2013 estimated that the prevalence of prediabetes in China was about 35.7% according to the diagnostic criteria of the American Diabetes Association (ADA) (4).

People who are diagnosed with diabetes require oral medications or insulin injections for life. According to International Diabetes Federation statistics, the average cost of diabetes treatments and disease management was about $3,219–$4,674 per diabetic patient annually in 2017 (5). Mean diabetes-related health expenditure per person (20–79 years) with diabetes in China was between $500 and $1,000 in 2019, which represents a substantial burden to the patients and their families (1). Diabetes also has a considerable impact on key aspects of the lives of the patients due to short-term and long-term complications, such as fatigue, frequent infections, vision loss, and kidney damage (6). Approximately 4.2 million adults are estimated to die as a result of diabetes and its complications in 2019 (1). Thus, job loss, frequent hospitalization, higher demand for medical and patient care, reduced social interactions, and worsening in lifestyle are some of the major problems that patients with diabetes have to face (7).

Considering the impact of diabetes on the physical, mental, and social activities of an individual, and in contrast to traditional biochemical indicators, morbidity and mortality, measuring health-related quality of life (HRQoL) of diabetes is being paid more and more attention from the perspective of the patient (8–12). HRQoL as a central domain of patient-based outcomes is essential for health decision-making (11). Many studies commonly used different scales to measure the quality of life, such as EuroQol-5D (EQ-5D), 36-item Short Form Health Survey (SF-36) or its shorter version the SF-12 (12 items), Audit of Diabetes Dependent Quality of Life (ADDQoL), Diabetes-Specific Quality of Life (DSQL), and so on (9).

The EQ-5D is one of the most feasible and widely used tools, which has been validated and used to assess the quality of life of individuals in chronic diseases, such as diabetes, chronic lung diseases, stroke, and chronic mental illnesses (7). Currently, three versions of EQ-5D are available, among which the 5-level EQ-5D version (EQ-5D-5L) was improved its sensitivity and reduce ceiling effects (7). SF-36 scale is relatively complex and can only be used after application and payment. At the same time, there is still no utility scoring system based on the Chinese population (13). ADDQoL, DSQL, and other specific scales for diabetes should not be used to evaluate the quality of life of populations with normal glycemic levels and prediabetes (14, 15).

Several studies using EQ-5D have shown the difference in HRQoL between patients with diabetes and the normal glycemic populations (6, 7, 7, 9, 15). A study in Germany showed that as blood glucose levels deteriorated, the HRQoL of older adults with NGT, prediabetes, and diabetes gradually reduced (16). However, there is a lack of data on the HRQoL of populations with normal glycemic levels, prediabetes, and diabetes in China or even a region in China.

The purpose of this study was to compare HRQoL among populations with normal glycemic levels, prediabetes, and diabetes in Southwest China and to identify factors associated with these three different blood glucose states. This study also aimed to offer baseline data that can be easily compared with other regions in China or across countries.

## Materials and Methods

### Ethics Approval and Consent to Participate

This study was reviewed and approved by the China Ethics Committee of Registering Clinical Trials, West China Hospital, Sichuan University (protocol number: ChiECRCT-20150048). Patients/participants provided written informed consent to participate in this study.

### Study Population

The survey was conducted in Sichuan province, southwest of China. Sichuan has a population of 82 million, accounting for 5.97% of the total population of China (16). Purposive and convenient sampling was used to select the participants of the three groups in this study. Patients with diabetes receiving treatments at the out-patient departments of several tertiary hospitals were selected, whose blood glucose levels were taken from their test sheets or querying the latest results within 3 months. The prediabetic group was selected from individuals who underwent physical examination in the tertiary hospitals and conformed to the inclusion criteria. People with normal glycemic levels were selected from individuals using the parks and community spaces who met the inclusion criteria. Before the investigators conducted a formal investigation, potential respondents were asked if they had diabetes or whether the value of their last blood glucose test was normal.

### Inclusion and Exclusion Criteria

Eligible diabetic participants fulfilled the following criteria: participated voluntarily; ≥18 years old; and were diagnosed by a physician or by asking whether they met the ADA diabetes diagnostic criteria (1), as follows: hemoglobin A1C (HbA1C) ≥ 6.5%, fasting plasma glucose (FPG) ≥ 126 mg/dl (7.0 mmol/L), or 2-h plasma glucose (PG) ≥ 200 mg/dl (11.1 mmol/L) during an oral glucose tolerance test (OGTT); or a patients with classic symptoms of hyperglycemia or hyperglycemic crisis, with random PG ≥ 200 mg/dl (11.1 mmol/L).

Eligible participants with prediabetes met the following criteria: the people participated voluntarily; ≥18 years of age; met the published ADA diagnostic criteria for prediabetes: FPG 100–125 mg/dl (5.6–6.9 mmol/L), 2-h PG during 75-g OGTT between 140 and 199 mg/dl (7.8–11.0 mmol/L) (IGT) or HbA1C 5.7–6.4%.

Eligible participants with normal glycemic levels fulfilled the following criteria: the people who participated voluntarily; ≥18 years old; with normal glycemic levels, or were free of self-reported diabetes.

Individuals who reported disturbance of consciousness and response, those with hearing loss or tinnitus, and those who were unable to fill out the questionnaire or had been diagnosed with gestational diabetes were excluded from all three study groups.

### Sample Size and Sampling

According to the sample size formula for simple random sampling:


$$\begin{array}{l} n=\;{\left(\frac{\mu_{\alpha /2}}{2\delta }\right)}^{2} \end{array}$$

**Figure 1:**
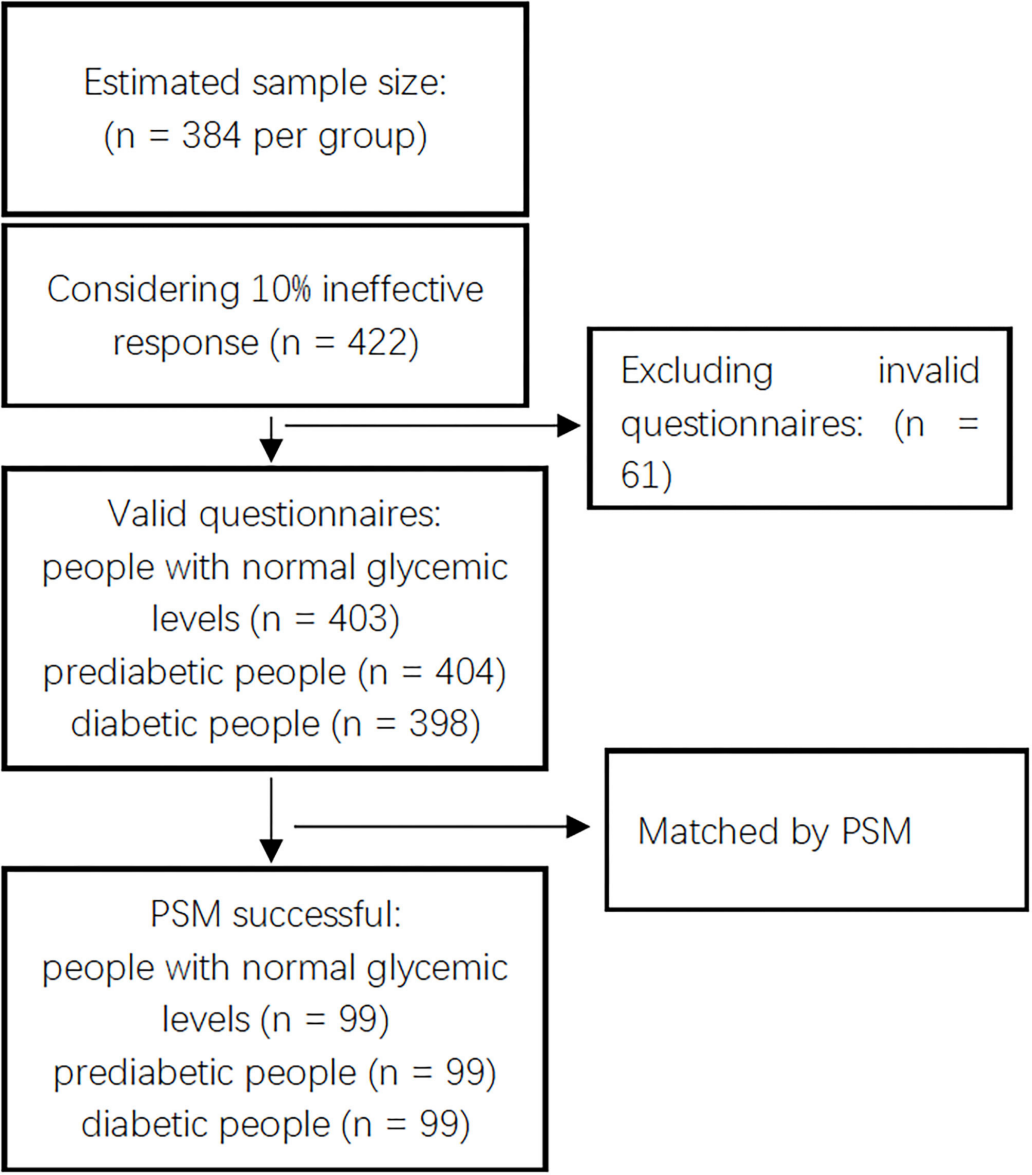
Study flowchart and participants recruited.

An adequate sample size was calculated for ≥384 participants. Accounting for a 10% ineffective response rate, we interviewed 422 people for each group and asked them to complete questionnaires. In this study, 403 effective questionnaires were collected from people with normal glycemic levels, 404 from individuals with prediabetes, and 398 from people with diabetes, after removing ineffective questionnaires, due to insufficient or incomplete data. The process of recruitment of participants is presented in Figure 1.

### Questionnaire Design

A draft questionnaire was designed based on a literature search and interviews with clinical pharmacists and endocrinologists. The questionnaire consisted of six parts: demographic data, disease state, economic state, health insurance, lifestyle, and interview EQ-5D-5L scale. A pilot survey that includes 20 interviewees was conducted in April 2015, based on the questionnaire was modified to include six parts with 22 questions. The EQ-5D-5L instrument was applied in this study after registering on www.euroqol.org. The EQ-5D-5L scale comprises a descriptive system and a EuroQol-visual analog scale (EQ-VAS). The descriptive system includes five dimensions: mobility, self-care, usual activities, pain/discomfort, and anxiety/depression. Each dimension was categorized into five levels: no problems, slight problems, moderate problems, severe problems, and extreme problems.

### Survey Implementation

Four trained investigators implemented the study from June 2015 to March 2016. Informed consent was obtained from each participant before they were surveyed. Participants with diabetes were interviewed using a face-to-face conversation method when they were waiting to see the doctor in the hospitals. People with normal glycemic levels were surveyed face-to-face in parks and community spaces. Respondents were first asked about their blood sugar status, based on their HbA1C level at the last physical examination. Those who did not know their blood glucose levels or who had diabetes were excluded. Questionnaires were completed by the investigators according to the description of the interviewee when they could not fully understand the questions. People with prediabetes were contacted *via* the hospital medical examination centers. Upon confirmation of the pre-diabetics status, surveys were conducted over the phone at an appointed time.

### Statistical Analysis

Questionnaire data were recorded using EpiData version 3.0 (EpiData Association, Odense, Denmark) and processed with Excel (2013). The reliability of the questionnaire was measured using Cronbach's alpha coefficient (α). The statistical package for social science (SPSS 20, Chicago, IL, USA) software was used for statistical analysis. Parametric tests were executed when the data followed a normal distribution, and non-parametric tests were selected when the data followed a non-normal distribution or did not meet the requirements for parametric tests. EQ-VAS scores and EQ-Index values are expressed as mean ± SD (x ± s). The EQ-Index was calculated using the Chinese EQ-5D-5L set (17). All statistical tests were two-sided. Testing the significance of differences and correlations between variables was conducted using the chi-square test, *t*-test, or one-way ANOVA. To decrease the bias introduced by *a priori* differences in the characteristics of participants in the three groups, we used propensity score matching (PSM) techniques, conditioned on age and gender, body mass index (BMI), and household income. As there were three groups, two groups were matched first, and the results then matched with the third group. The three groups were directly compared to explore HRQoL differences among them.

The study flowchart and participants recruitment are presented in Figure 1.

## Results

### Sociodemographic Characteristics

The Cronbach's α scores for the diabetic, prediabetic, and normal glycemic levels groups were 0.721, 0.748, and 0.753, respectively, and the content validity values were 0.673, 0.728, and 0.840, respectively. These data demonstrate that the questionnaire showed good reliability and validity (18). There were significant differences in age, BMI, sex, alcohol consumption, education level, type of medical insurance, household income, and family history of diabetes among the three groups of respondents. The sociodemographic features of respondents, grouped by glucose status, are presented in Table 1.

**Table 1:** Sociodemographic features of respondents grouped by glucose status.

**Variable**	**Normal glycemic level** **(*N* = 403)**	**Pre-diabetic** **(*N* = 404)**	**Diabetic** **(*N* = 398)**	***P-*value**
Mean age [years (SD)]^*^	58.94 (12.48)	53.29 (9.21)	62.23 (11.3)	<0.001
**Male sex [*****n*** (%)]^*^	195 (48.39%)	246 (60.89%)	212 (53.27%)	<0.001
**BMI [kg/m**^**2**^ (SD)]^*^	23.67 (4.24)	23.73 (2.99)	24.57 (4.05)	0.001
**Cigarette smoking**				0.653
Occasionally	30	37	20	
Often	96	100	117	
Never	277	267	261	
**Number of cigarettes smoked per day**				0.269
None	276	266	260	
<5 per day	22	37	15	
5–10	29	37	24	
10–20	47	37	46	
>20	29	27	53	
Alcohol consumption^*^				0.002
Occasionally	80	120	71	
Often	49	43	55	
Never	274	241	272	
**Approximate amount of alcohol consumption per day (ml)**				0.400
None	273	241	272	
50	69	130	53	
100	35	22	28	
150	8	6	12	
200	18	5	33	
Education level^*^				<0.001
Primary and below	132	27	134	
Junior or high school	164	106	124	
College graduated	56	132	80	
Master's degree and above	51	139	60	
Health insurance^*^				<0.001
None	8	2	5	
China's urban employee basic medical insurance	137	347	151	
China's new rural cooperative medical system	157	36	69	
China's medical insurance for urban residents	94	19	78	
Other commercial insurance	7	0	95	
Household income^*^				<0.001
Low	85	8	50	
Average	178	80	157	
Relatively high	121	163	139	
High	19	153	52	
**Sleep status**				0.214
Very poor	31	13	12	
Poor	87	75	106	
General	131	167	113	
Good	144	117	147	
Very good	10	32	20	
Family history of diabetes^*^				<0.001
None	351	311	269	
Yes	52	93	129	

* *Difference was statistically significant*.

### Blood Glucose Status

Blood glucose data were gathered from prediabetic and diabetic participants (Table 2). It should be noted that blood glucose levels in diabetic participants were recorded following the use of diabetic medications. The glucose levels of respondents with normal glycemic levels were not recorded; therefore, accurate glucose data cannot be provided. According to the *Guidelines for Prevention and Treatment of Hypertension in China*, the standard for well-controlled blood glucose is to maintain FPG levels between 3.9 and 7.2 mmol/L, 2-h PG ≤ 10 mmol/L, and HbA1c <7%. The rates of the well-controlled FPG, 2-h PG and HbA1c among participants with diabetes were 40.0, 31.03, and 40.58%, respectively.

**Table 2:** Composition and blood glucose status of the three groups.

**Disease types**	**Disease categories**	**Normal glycemic**		**Prediabetics**		**Diabetics**	
		**Number**	**Constituent ratio**	**Number**	**Constituent ratio**	**Number**	**Constituent ratio**
Co-morbidity	Circulatory system diseases (hypertension, Cardiovascular disease, etc.)	102	25.31%	94	23.27%	152	38.19%
	Nutritional metabolic diseases (hyperlipidemia, fatty liver, etc.)	42	10.42%	90	22.28%	125	31.41%
	Other diseases (arthritis, protrusion of intervertebral disc, etc.)	13	3.23%	11	2.72%	40	10.05%
	Digestive system diseases (gastritis, cholecystitis, etc.)	10	2.48%	20	4.95%	33	8.29%
	Urinary system diseases (urinary calculi, cholelithiasis, etc.)	2	0.50%	0	0.00%	16	0.00%
	Hematological diseases (cerebral ischemia)	6	1.49%	5	1.24%	11	2.76%
	Endocrine system diseases (thyroid disease)	9	2.23%	5	1.24%	5	1.26%
	Respiratory diseases (emphysema, pneumonia, etc.)	10	2.48%	3	0.74%	2	0.50%
	Nervous system diseases (deafness)	1	0.25%	0	0.00%	2	0.50%
Complications	Other diseases (diabetic foot, diabetic retinopathy, etc.)	0	0.00%	0	0.00%	85	21.36%
	Urinary system diseases (diabetic nephropathy)	3	0.74%	4	0.99%	16	4.02%
HbA1c (%) (x ± SD)		–		5.93 ± 0.31		7.56 ± 2.00	
FPG (mmol/L) x ± SD		–		6.35 ± 0.73		8.16 ± 2.98	
2hPG (mmol/L) x ± SD		–		9.17 ± 1.73		8.16 ± 2.98	

### Complications of Diabetes

Previous studies have shown that circulatory and nutritional and metabolic diseases are the most common complicating diseases in all three groups and that the incidence of complications increases with an elevated risk of diabetes. Our results showed that the occurrence rate of complications was 38.19% in patients with diabetes with circulatory system diseases and 31.41% in those with nutritional and metabolic diseases. The occurrence rate of complications was 23.27% in patients with prediabetes with circulatory system diseases and 22.28% in those with nutritional and metabolic diseases, while it was 25.31% in people with normal glycemic levels with circulatory system diseases and 10.42% in those with nutritional and metabolic diseases. More than half of people with diabetes had other co-morbid diseases in this research. Hypertension, hyperlipidemia, and diabetic retinopathy ranked as the top three among all the complications (Table 2).

### Quality of Life Measurement

**Figure 2:**
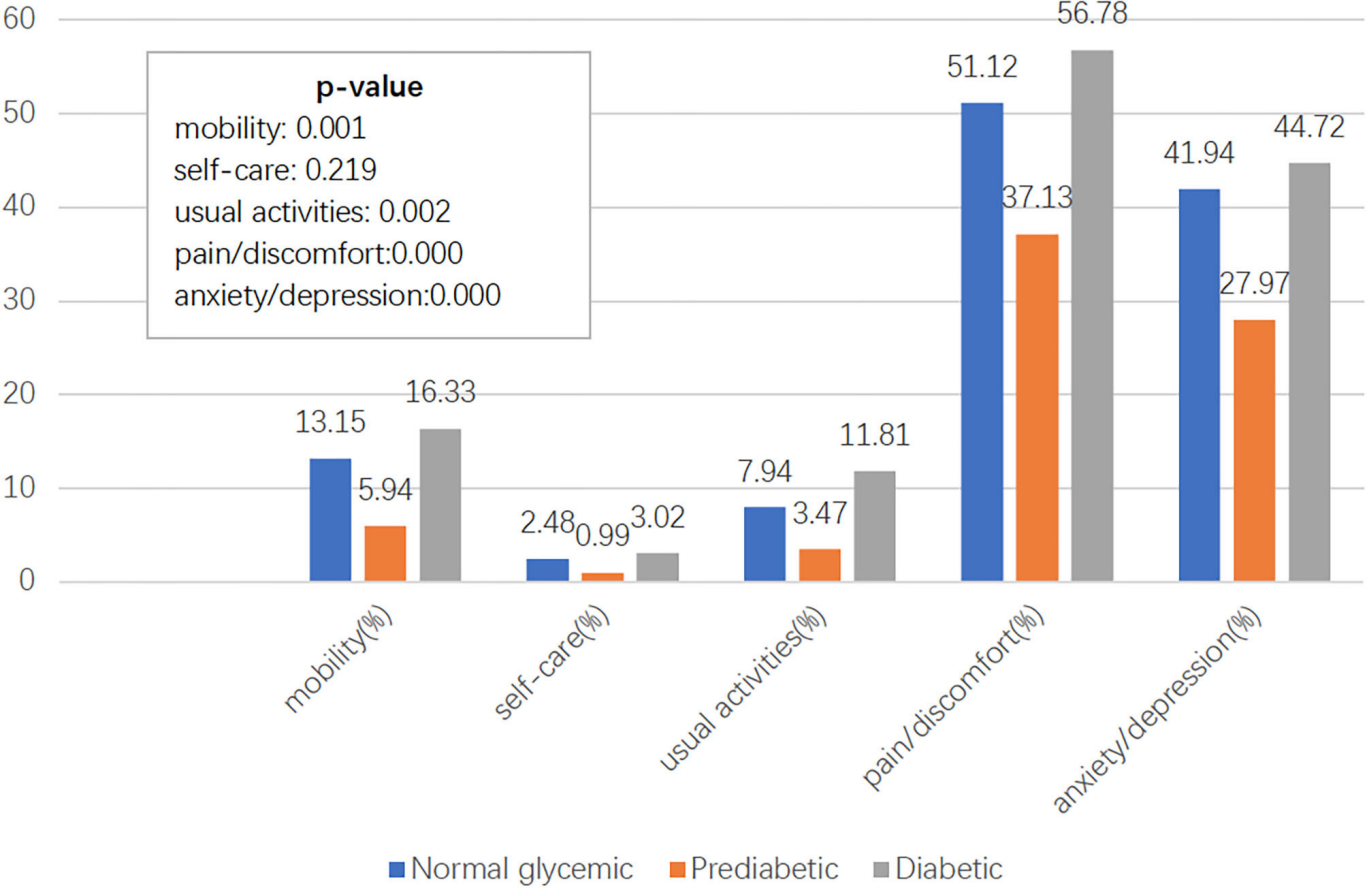
Percentage of self-rated problematic on five dimensions.

As shown in Figure 2, the main health-related problems of ED-5D reported by respondents with different glycemic levels were pain/discomfort, anxiety/depression, and difficulties with self-care, which had the lowest ranking. The proportions of health-related problems in the five dimensions were consistent among the three groups. Health-related problems self-assessed by respondents ranked from high to low are as follows: pain/discomfort, anxiety/depression, mobility, usual activities, and self-care.

**Figure 3:**
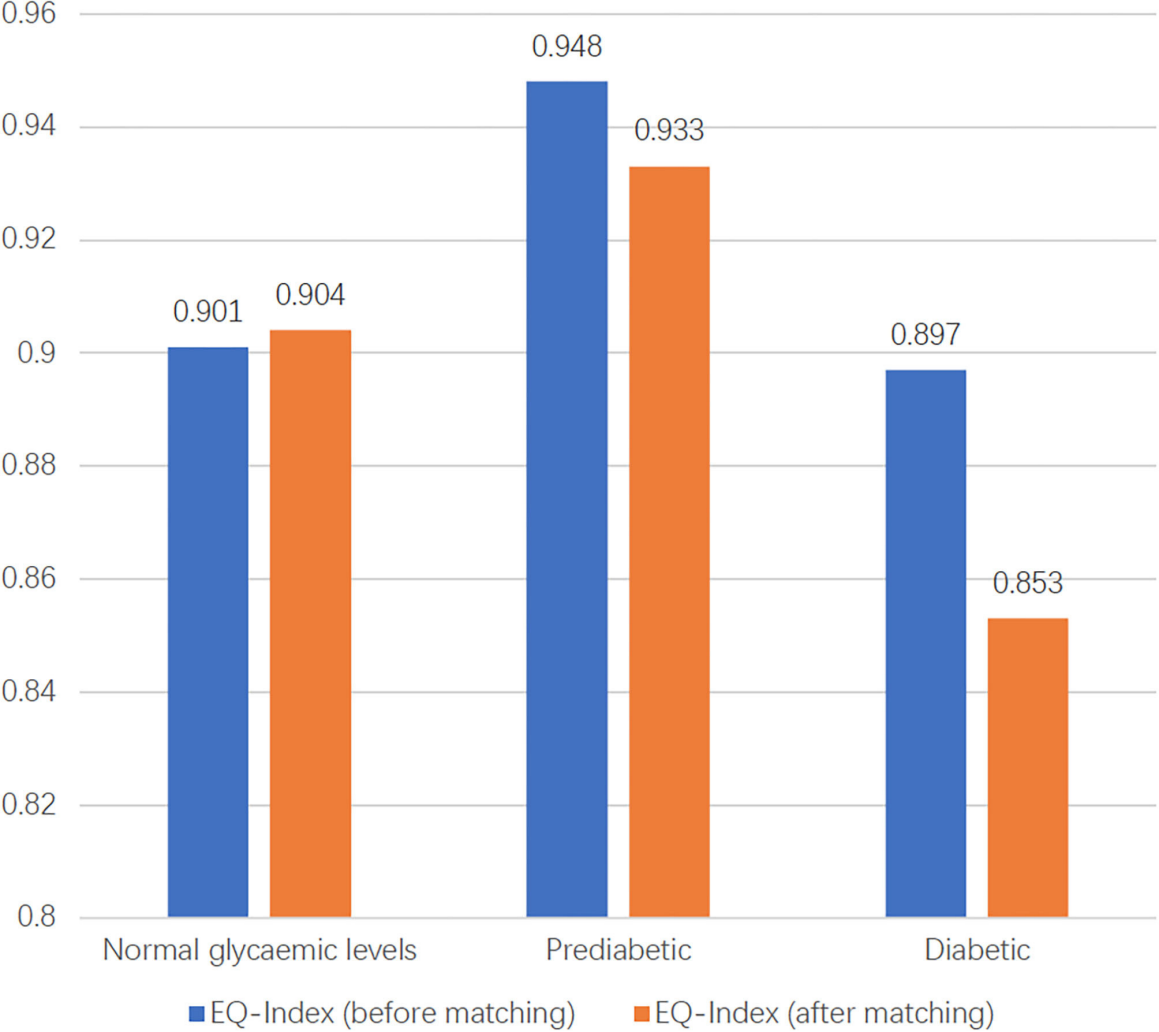
The distribution of EQ-Index of three groups before and after matching.

**Figure 4:**
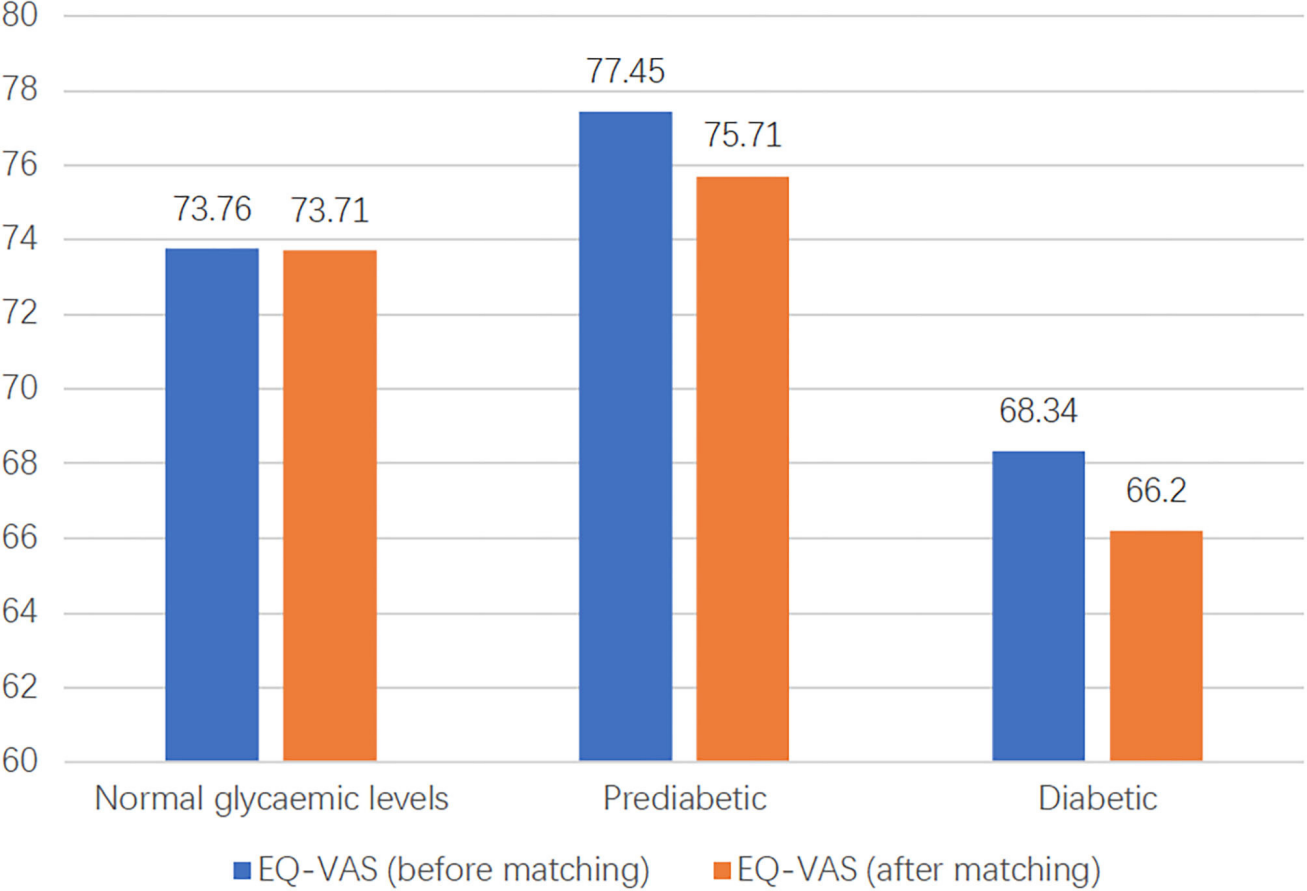
The distribution of EQ-VAS of three groups before and after matching. EQ-VAS, EuroQol-visual analog scales.

EuroQol visual analog scale score and EQ-Index of people with diabetes, prediabetics, and normal glycemic levels are presented in Figures 3, 4. The highest EQ-VAS scores were recorded for the group with prediabetes, followed by those with normal blood glucose and then patients with diabetes. In this study, EQ-Index values were calculated using the Chinese EQ-5D-5L value sets and processed by the dimension reduction method. People with diabetes had lower health utility values than those with normal glycemic levels, while individuals with prediabetes had higher health utility values than those with normal glycemic levels.

To compare the differences in HRQoL among the three glucose status populations, PSM was used to match participants according to age, income, BMI, and sex. As there were three groups, we matched two groups first and then matched the results with the third group. Finally, 99 individuals from each group were matched. The results of PSM showed that the EQ-VAS values of the populations with normal glycemic levels, prediabetes, and diabetes were 73.71, 75.71, and 66.20, and EQ and EQ-Index of the three groups were 0.904, 0.933, 0.853, respectively (Figures 3, 4). Hence, the population with prediabetes had the highest EQ-VAS values, followed by those with normal glycemic levels and those with diabetes, which was consistent with the result before matching. After matching, the EQ-Index values of the three groups had the same trend as before matching; however, there was no significant difference among the three groups.

## Discussion

This study described the HRQoL of populations with normal glycemic levels, prediabetes, and diabetes in southwest China. Compared with EQ-VAS scores ranging from 61 to 79 in other EQ-5D surveys, this study found a score of 69.35 for southwest Chinese diabetics, which is at an intermediate level, relative to previous reports. Compared with the data from other countries, the mean EQ-VAS scores of the southwest Chinese diabetic population were slightly higher than that of Spain and Poland, which were 61.106 and 68.2, respectively (19, 20). The EQ-Index values determined in this study were consistent with those reported by Zhou et al. (21) for Chinese patients with diabetes range, 0.79–0.94, indicating that the results of this study were still in the middle of this range. Compared with the EQ-Index scores of people with diabetes reported from in Spain (19) and German (22) of 0.742 and 0.80, respectively, the scores for individuals with diabetes in this study were slightly higher.

Further, the prevalence rates of diabetes and prediabetes were higher among males than females in this study; however, the differences were not significant. Nevertheless, our results are consistent with those of other reports that the prevalence of diabetes in females is lower than that in males in China (23–25); hence, it is particularly important for men to pay attention to their PG levels and relevant lifestyle factors.

Our study showed that half of the patients with diabetes exhibited complications: 32.25% had one type of complication, 19.50% had two types of complications, and about 11.00% had more than three types of complications. Among all complications, macroangiopathy and microangiopathy were the main causes of disability and death in older patients with diabetes. In this study, atherosclerosis accounted for 0.75% of patients with diabetes with macrovascular disease, while the incidence rates of retinopathy, neuropathy, and anemia were 9.25, 4.75, and 0.5%, respectively. Therefore, patients with diabetes in southwest China should aim to prevent macroangiopathy and microangiopathy, particularly, atherosclerosis, retinopathy, neuropathy, and anemia.

In our survey, 52% of patients had taken action to control their diet and exercise, while only 14% of patients took such actions continuously. Most patients misunderstood how to control their diet and exercise. For dietary intervention, most patients avoided high sugar and high-fat foods, as a general guideline; however, they lacked awareness of calculating calories in foods. Therefore, education of the patients about diet may be necessary.

The guideline for the prevention and treatment of type 2 diabetes mellitus in China (2020 edition) suggests that the long-term treatment goals of diabetes should include improving the quality of life of patients (26). However, at present, there is no unified method and clear judgment criteria for measuring the quality of life of diabetes in clinical management. The results of our study could provide a reference or baseline for HRQoL assessment of diabetes. In this area, numerous studies reported that pain/discomfort and anxiety/depression are the major complaints by the diabetes (27, 28). Our study compared the three groups and found that pain/discomfort and anxiety/depression were also the influencing factors of quality of life in the population with normal glycemic levels and prediabetes. This result suggested that pain/discomfort and anxiety/depression might not be specific for the population with or without diabetes. A more specific index needs to be optimized or developed.

The results of our survey indicate that the quality of life in participants with prediabetes was higher than that of those with normal glycemic levels and diabetes. This result differs from the findings of a previous German survey, which showed that the quality of life gradually decreased as glycemic status deteriorated (22). This may be due to some design flaws in our study. Samples were collected from three different populations, and prediabetic group samples were primarily from people who attended the physical examination in tertiary hospitals and likely had better awareness and self-care ability. Simultaneously, most of the population had good jobs, access to health insurance, advantages related to family income and educational background, and advantages related to the groups with diabetes and normal glycemic levels. Therefore, it was easier for participants with prediabetes to maintain good physiological and mental states.

Our study had limitations, such as the influence of recall bias on the blood sugar indicators, diet, exercise, sleep, and illness data acquired using the questionnaire. Further, the study design was cross-sectional and, therefore, cannot directly determine the causal relationships between changes in quality of life and the dynamic evolution of diabetes. Furthermore, we did not discuss the EQ-VAS scores of subgroups of patients with diabetes, due to the large differences in sample sizes among them. Finally, this study was limited to Sichuan, southwest of China, and differences in lifestyles may have influenced our findings.

## Conclusion

We found a general trend of a decline in HRQoL of the patient from the prediabetic population, to the population with normal glycemic levels, and then the diabetic population in southwest China. Further and larger longitudinal studies are needed to confirm these findings. Pain/discomfort and anxiety/depression are the influencing factors of quality of life among three groups, which might not be specific for the population with or without diabetes.

## Data Availability Statement

The raw data supporting the conclusions of this article will be made available by the authors, without undue reservation.

## Ethics Statement

The studies involving human participants were reviewed and approved by China Ethics Committee of Registering Clinical Trials, West China Hospital, Sichuan University (protocol number: ChiECRCT-20150048). The patients/participants provided their written informed consent to participate in this study.

## Author Contributions

EL contributed to data acquisition, interpretation of data, and revised the manuscript critically. NY contributed to research design, data analysis/interpretation, and wrote the manuscript. SF, JC, and LZ contributed to data acquisition, data analysis/interpretation, and revised the manuscript. LS and XJ contributed to the statistical analysis and reviewed the manuscript. MH contributed to research design, data analysis/interpretation of data, and revised the manuscript critically. All authors read and approved the final manuscript.

## Conflict of Interest

The authors declare that the research was conducted in the absence of any commercial or financial relationships that could be construed as a potential conflict of interest.

## Publisher's Note

All claims expressed in this article are solely those of the authors and do not necessarily represent those of their affiliated organizations, or those of the publisher, the editors and the reviewers. Any product that may be evaluated in this article, or claim that may be made by its manufacturer, is not guaranteed or endorsed by the publisher.

## Abbreviations

NGT: normal glucose tolerance
ADA: American Diabetes Association
HRQoL: health-related quality of life
EQ-5D: EuroQol-5D
SF-36: 36-item Short Form Health Survey
ADDQoL: Audit of Diabetes Dependent Quality of Life
DSQL: Diabetes-Specific Quality of Life
BMI: body mass index
EQ-5D-5L: EuroQoL-5 Dimension-5 levels
EQ-VAS: EuroQol-visual analog scales
FPG: Fasting Plasma Glucose
HbA1C: Hemoglobin A1C
OGTT: oral glucose tolerance test
PG: plasma glucose
PSM: propensity score matching.
